# LPCAT1 acts as an independent prognostic biomarker correlated with immune infiltration in hepatocellular carcinoma

**DOI:** 10.1186/s40001-022-00854-1

**Published:** 2022-10-28

**Authors:** Lan Li, Xiao Wang, Yanni Ding, Nini Hui, Bingjie Su, Min Yang

**Affiliations:** Department of Breast Surgery, Shaanxi Provincial Cancer Hospital, Xi’an, 710061 Shaanxi China

**Keywords:** LPCAT1, Prognosis, Tumor microenvironment, Immune cell infiltration, Immune checkpoint, Drug sensitivity, Signaling pathway, Hepatocellular carcinoma

## Abstract

**Background:**

Lysophosphatidylcholine acyltransferase 1 (LPCAT1) is overexpressed in multiple human tumors. However, the role of LPCAT1 in hepatocellular carcinoma (HCC) has not been understood. We aim to explore the relationships between LPCAT1 expression and prognosis, clinicopathological features, tumor microenvironment (TME), immune cell infiltration, immune checkpoint gene expression, and related signaling pathways in HCC. Furthermore, we also explored the relationship between LPCAT1 expression and drug sensitivity to HCC treatment.

**Methods:**

The expression profiles were acquired from the Cancer Genome Atlas (TCGA) and the Human Protein Atlas (THPA). Immune status and infiltration in cancer tissues were explored using the single sample gene set enrichment analysis (ssGSEA) and CIBERSORT algorithm.

**Results:**

LPCAT1 was overexpressed in HCC, and its expression was related to poor prognosis, LPCAT1 was an independent prognostic biomarker in HCC. Expression of LPCAT1 increased statistically with the increase of clinical stage and grade of HCC patients. GO and KEGG network analysis revealed that LPCAT1 positively associated molecules were mostly enriched in functions related to cell adhesion. The TME score of high-LPCAT1 group was significantly higher than that of low-LPCAT1 group. Immune infiltrating cells positively correlated with LPCAT1 expression were Macrophages M0, B cells memory, Dendritic cells activated, T cells regulatory and T cells gamma delta in HCC. We found a positive correlation between LPCAT1 and most immune checkpoint gene expression. The IC50 of 5-Fluorouracil, Gemcitabine, Mitomycin C, Sorafenib and Cabozantinib in patients with high-LPCAT1 expression was lower than that in patients with low-LPCAT1 expression. Our findings provide a wealth of information for further understanding of the biological functions and signaling pathways of LPCAT1 in HCC.

**Conclusions:**

LPCAT1 is an independent prognostic biomarker and associated with tumor microenvironment, immune cell infiltration, immune checkpoint expression and drug sensitivity in hepatocellular carcinoma.

## Background

HCC is the most frequent type of malignant liver tumor worldwide, with a complex etiology, insidious onset and high malignancy [1]. Currently, chemotherapy is still an effective treatment for liver cancer. Molecular-based targeted therapies are also increasingly becoming systemic treatments for liver cancer, including Sorafenib (RAF/MEK/ERK signaling pathway inhibitor, VEGFR and PDGFR inhibitor), Lenvatinib (VEGFR-1, VEGFR-2, VEGFR-3, FGFR1, PDGFR, cKit and Ret inhibitor), Bevacizumab (VEGF inhibitor) and other monoclonal antibodies. Despite appreciable progress has been achieved in systematic treatments, the overall long-term survival of HCC patients is still unsatisfying [2]. Therefore, it is of great significance to predict the prognosis of patients with HCC and search for therapeutic targets.

Metabolic pathways, including lipid metabolism, are frequently reprogrammed within cancer cells to adapt to environmental challenges and promote their expansion and metastasis [3–5]. As a member of LPCATs family, LPCAT1 is an enzyme involved in phosphatidylcholine metabolism and is essential for the regulation of phosphatidylcholine composition [6, 7], especially for the accumulation of polyunsaturated fatty acids [8]. There is increasing evidence that the expression of LPCAT1 is overexpressed in multiple human tumors and may be involved in tumor proliferation and metastasis [9–11]. However, there is less in-depth information about LPCAT1 in HCC, which needs further study.

In our study, we analyzed the relationship between LPCAT1 expression and clinicopathological features and prognosis in HCC. The correlation of LPCAT1 expression with TME score, immune cell infiltration and immune checkpoint gene expression were further studied. Moreover, biological functions and related signaling pathways of LPCAT1 was explored in HCC. Finally, we explored the relationship between LPCAT1 expression and drug sensitivity to HCC treatment.

## Methods

### LPCAT1 expression analysis

All available data of 21 cancer types were downloaded from TCGA as Fragments per kilobase per million (FPKM), and then, the data were transformed into transcripts per million (TPM). Finally, Mann Whitney *U* test was performed using the “ggplot2” R package. The immunohistochemistry of LPCAT1 was obtained from THPA, a program which aims to map all the human proteins in cells, tissues and organs using integration of various omics technologies [12]. This study complies with the publication guidelines and access rules of TCGA.

### Characteristic comparison between the high- and low-LPCAT1 groups

Clinical data from TCGA were used to analyze the overall survival and progression-free survival of HCC patients between 2 groups. Clinical characteristics including Age, Gender, Grade, Stage, T, M, and N were included to analyze whether there were differences between 2 groups. “ggplot2” R package was used to analyze differential genes expression between 2 groups. General immune status of each patient was assessed by ESTIMATE score, stromal score, immune score and tumor purity score. In addition, in each HCC sample, we utilized CIBERSORT algorithm and depicted the profile of 22 immunocyte subtypes [13]. The obtained data were analyzed within high- and low-LPCAT1 groups by ssGSEA with the “GSVA” R package [14, 15]. “pRRophetic” R package was used to predict IC50 of 5-Fluorouracil, Gemcitabine, Mitomycin C, Sorafenib, Cabozantinib and Irinotecan in each sample. IC50 indicates the effectiveness of a substance in inhibiting specific biological or biochemical functions.

### GO and KEGG

GO is a database established for the definition and description of gene and protein functions applicable to various species. KEGG is a comprehensive database that integrates genomic, chemical, and systemic functional information. The enrichment analysis of LPCAT1-related genes was performed by the “clusterProfiler” R package [16] (v. 3.14.3) of R software (v. 3.6.3), and the results were visualized by the “ggplot2” R package (v. 3.3.3). The threshold conditions are set as: *p*.adj < 0.05 and *q *value < 0.2.

### Statistical analysis

In this study, Student’s *t* test was used to compare continuous variables and Mann–Whitney *U* test was used for categorical variables. The prognostic value of LPCAT1 was evaluated by univariable and multivariable analysis, which were further evaluated by Kaplan–Meier analysis. Spearman’s value was used to evaluate the correlation of LPCAT1 with checkpoint gene expression in HCC, and visualized by “ggplot2” R package (v. 3.3.3). R software was utilized to construct the heatmaps, forest plots, and circle plot. *p* < 0.05 was set as statistically significant. All statistical analyses were conducted by R software (v.4.1.3).

## Results

### Pan-cancer analysis of LPCAT1 expression

**Fig. 1:**
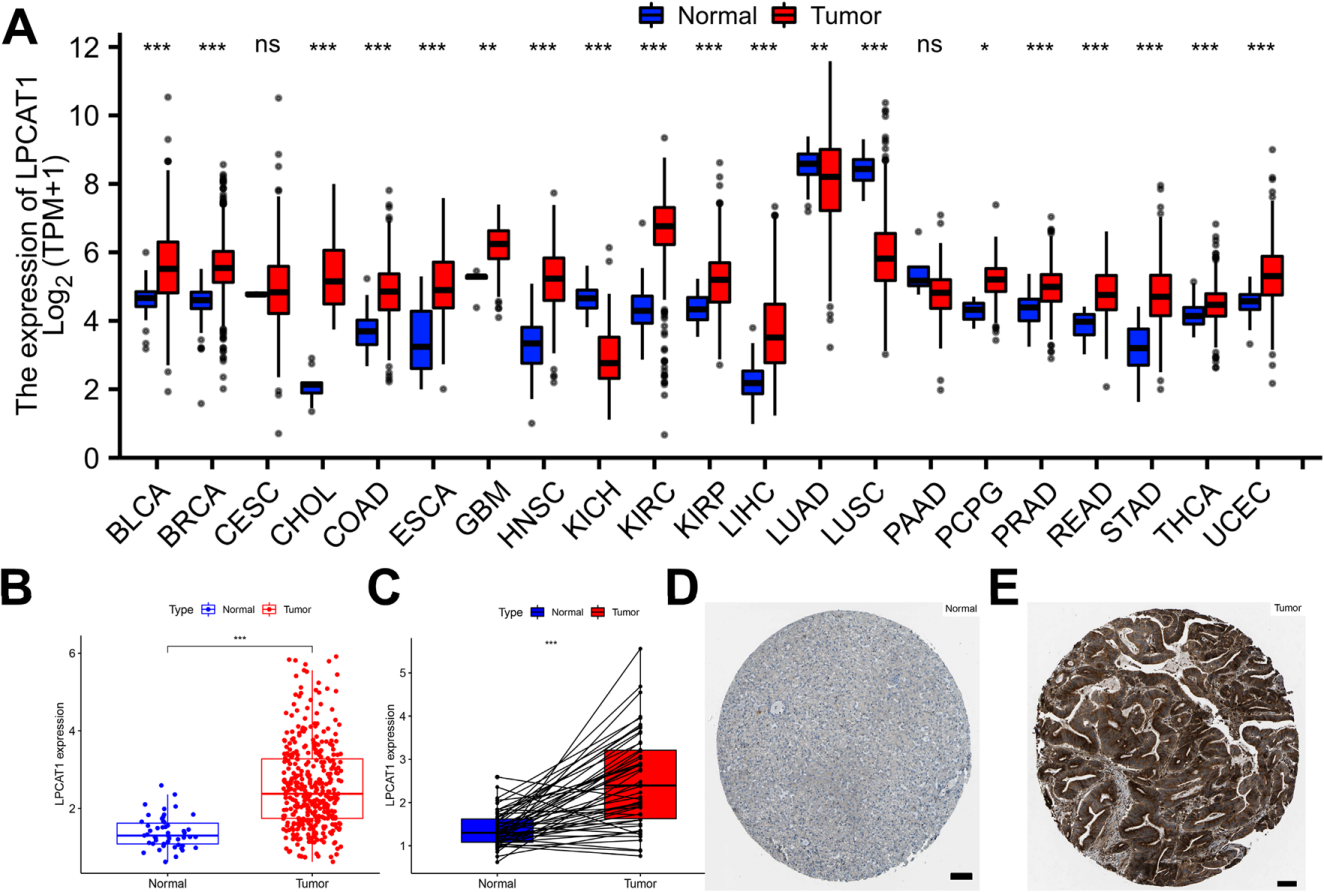
Pan-cancer analysis of LPCAT1 expression in human cancers. **A** LPCAT1 expression in human cancers compared with normal determined by TCGA. **B** LPCAT1 expression in HCC and normal tissues (unpaired samples). **C** LPCAT1 expression in HCC and normal tissues (paired samples). **D**, **E** Immunohistochemistry of LPCAT1 in HCC and normal tissues. ns: no significance; *p value < 0.05; **p value < 0.01; ***p value < 0.001. Scale is 100 μm

To determine the expression of LPCAT1 in human tumors, we first performed its expression analysis in 21 human tumors. As shown in Fig. 1A, our results showed that LPCAT1 was overexpressed in 16 cancer types, including BLCA, BRCA, CHOL, COAD, ESCA, GBM, HNSC, KIRC, KIRP, LIHC, PCPG, PRAD, READ, STAD, THCA and UCEC, and significantly decreased in KICH, LUAD and LUSC. In addition, we found no significant differences in the expression of LPCAT1 in CESC and PAAD. We further created the scatter plot of LPCAT1 expression in HCC and normal controls, as shown in Fig. 1B, LPCAT1 expression was significantly increased in HCC compared with normal tissues, with statistical significance. The results of paired sample analysis also showed that the expression of LPCAT1 was significantly increased in HCC (Fig. 1C). Finally, immunohistochemical results of THPA confirmed above results (Fig. 1C, D). Taking all into consideration, the expression of LPCAT1 was overexpressed in 14 tumors: BLCA, BRCA, CHOL, COAD, ESCA, HNSC, KIRC, KIRP, LIHC, PRAD, READ, STAD, THCA, and UCEC, suggesting that LPCAT1 may play a key promoting role in the development of these 14 cancers including HCC.

### Prognostic value of LPCAT1 in HCC

**Fig. 2:**
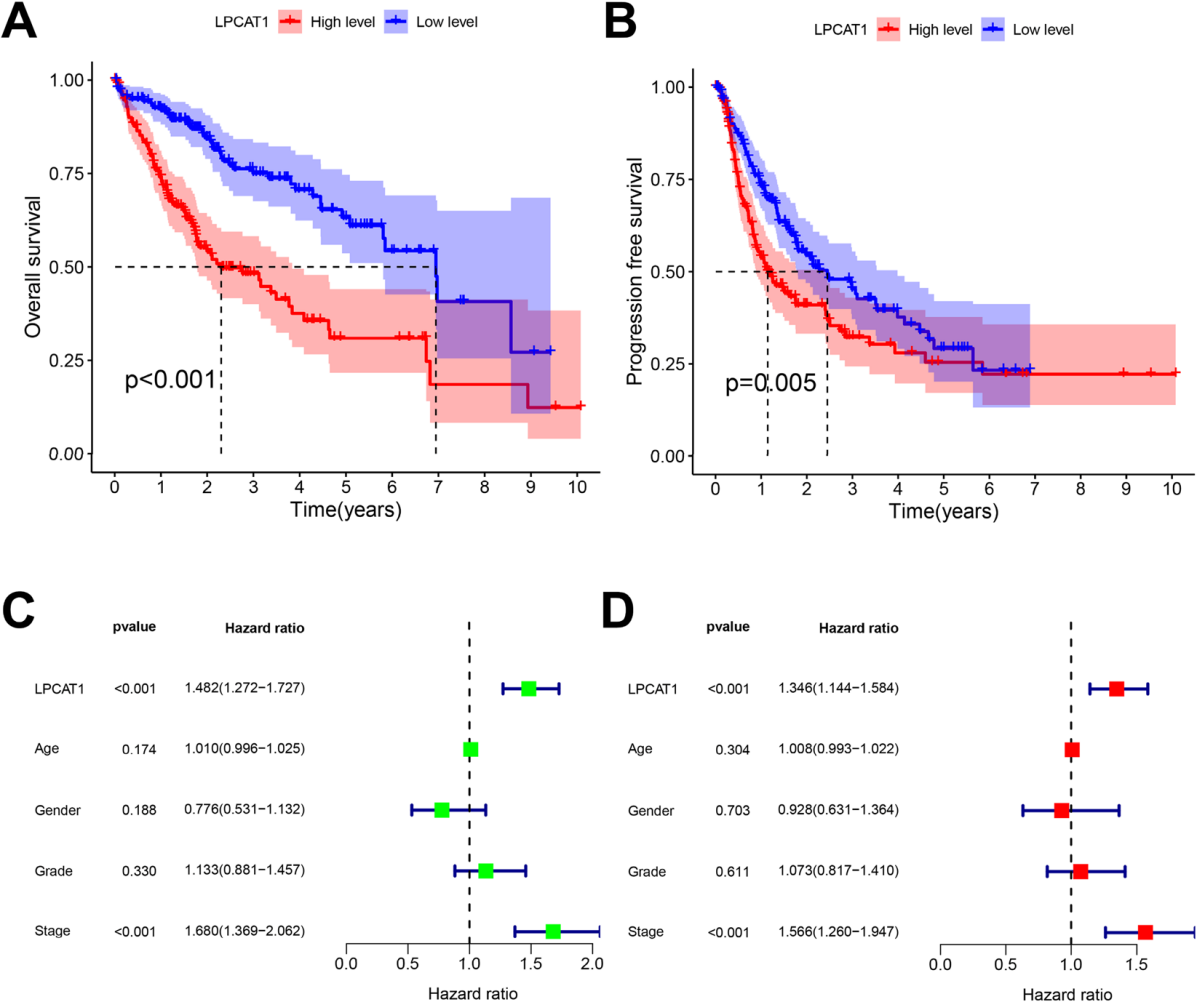
Prognostic value of LPCAT1 expression in HCC. Kaplan–Meier curves of overall survival **A** or progression free survival **B** in high- or low-LPCAT1 groups. **C**, **D** Univariable and multivariable analyses of LPCAT1 and various clinical factors

Next, we performed the prognosis analysis of LPCAT1 expression in HCC. As shown in Fig. 2A, B, the OS and PFS of patients with low-LPCAT1 were significantly better than those of patients with high-LPCAT1, indicating that high-LPCAT1 expression predicts poor prognosis. To determine whether LPCAT1 is an independent prognostic factor in HCC patients, we performed univariate/multivariate analysis, including LPCAT1, Age, Gender, Grade and Stage. Univariate analysis showed that LPCAT1 was negatively correlated with the prognosis of HCC patients, with statistical significance (Fig. 2C, OS: HR = 1.482, CI   1.272–1.727, *p* < 0.001). Further multivariate analysis showed that LPCAT1 was an independent prognostic factor in HCC patients (Fig. 2D, OS: HR = 1.346, CI   1.144–1.584, *p* < 0.001). These results demonstrated the prognostic values of LPCAT1 expression in HCC and can be used as an independent prognostic biomarker for HCC patients.

### Correlation of LPCAT1 expression with clinicopathological features in HCC patients

**Fig. 3:**
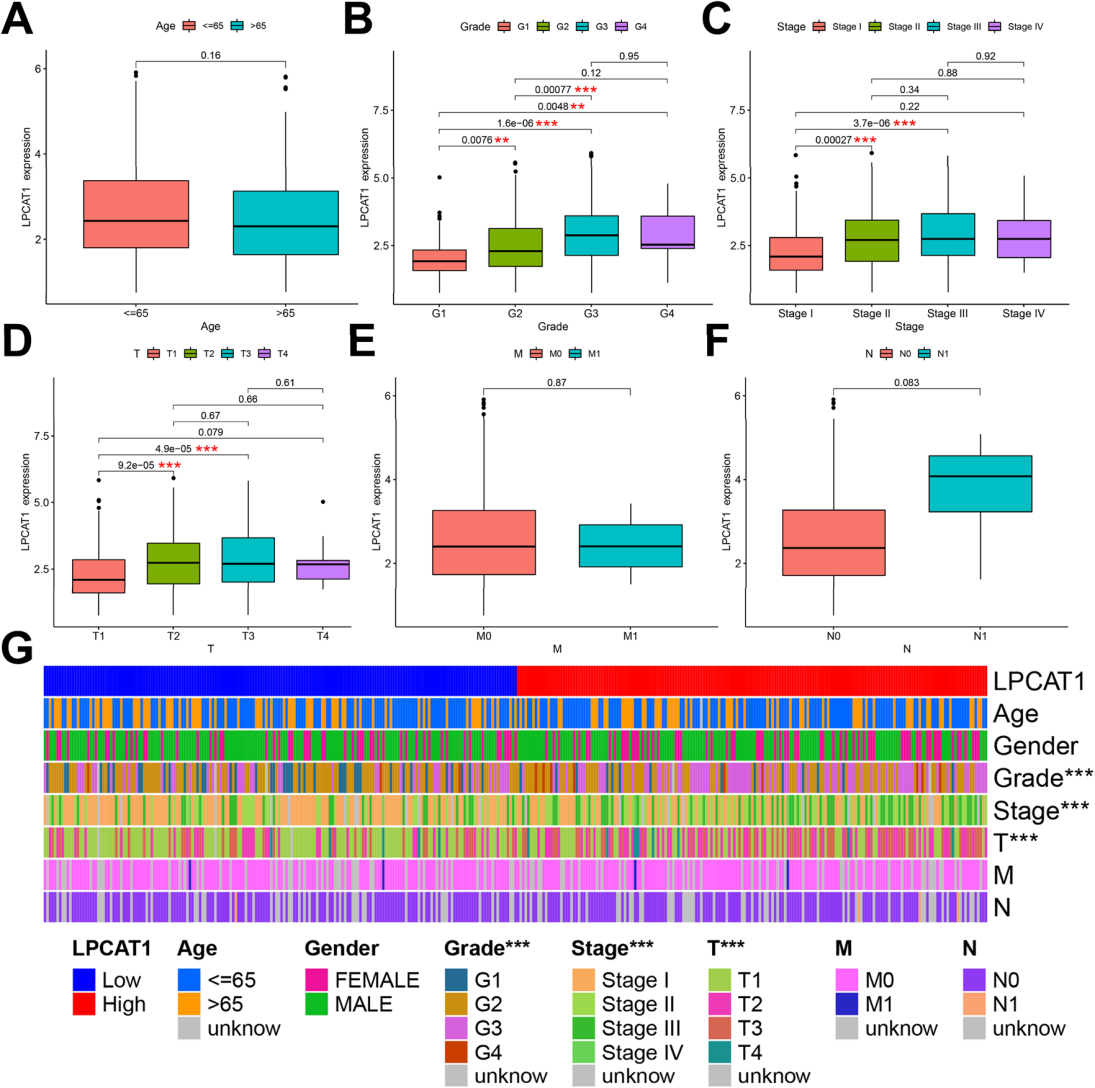
Correlation between LPCAT1 and clinicopathological features in HCC. Correlation of LPCAT1 expression in different groups classified by age (**A**), grade (**B**), stage (**C**), T (**D**), M (**E**) and N (**F**). **G** Heatmap of clinicopathological correlations with high- or low-LPCAT1 groups. *p value < 0.05; **p value < 0.01; ***p value < 0.001

Here, we used TCGA to further explore the correlation between LPCAT1 expression and clinicopathological features. As shown in Fig. 3B–D, we found that the expression of LPCAT1 increased statistically with the increase of clinical stage and grade of HCC patients (Grade: G1 vs G2, *p* = 7.6e−03; G1 vs G3, *p* = 1.6e−06; G1 vs G4, *p* = 4.8e−03; G2 vs G3, *p* = 7.7e−04; Stage: StageI vs Stage II, *p* = 2.7e−04; StageI vs Stage III, *p* = 3.7e−06; T: T1 vs T2, *p* = 9.2e−05; T1 vs T3, *p* = 4.9e−05). We didn’t find any relationship between LPCAT1 expression and Age, M and N (Fig. 3A, E, F). To clarify which clinicopathological features were different within high- and low-LPCAT1 groups, we created a heatmap of clinical correlations. The results are shown in Fig. 3G, the three clinicopathological features of Grade, Stage and T were significantly different between high- and low-LPCAT1 groups, with statistical significance (*p* < 0.001). We elucidated the correlation of LPCAT1 expression with clinicopathological features of HCC in detail. Together, our results demonstrated that LPCAT1 expression is positively correlated with the risk of HCC, which can well reflect the clinical stage and grade of HCC patients.

### Analysis of biological functions and related signaling pathways of LPCAT1

**Fig. 4:**
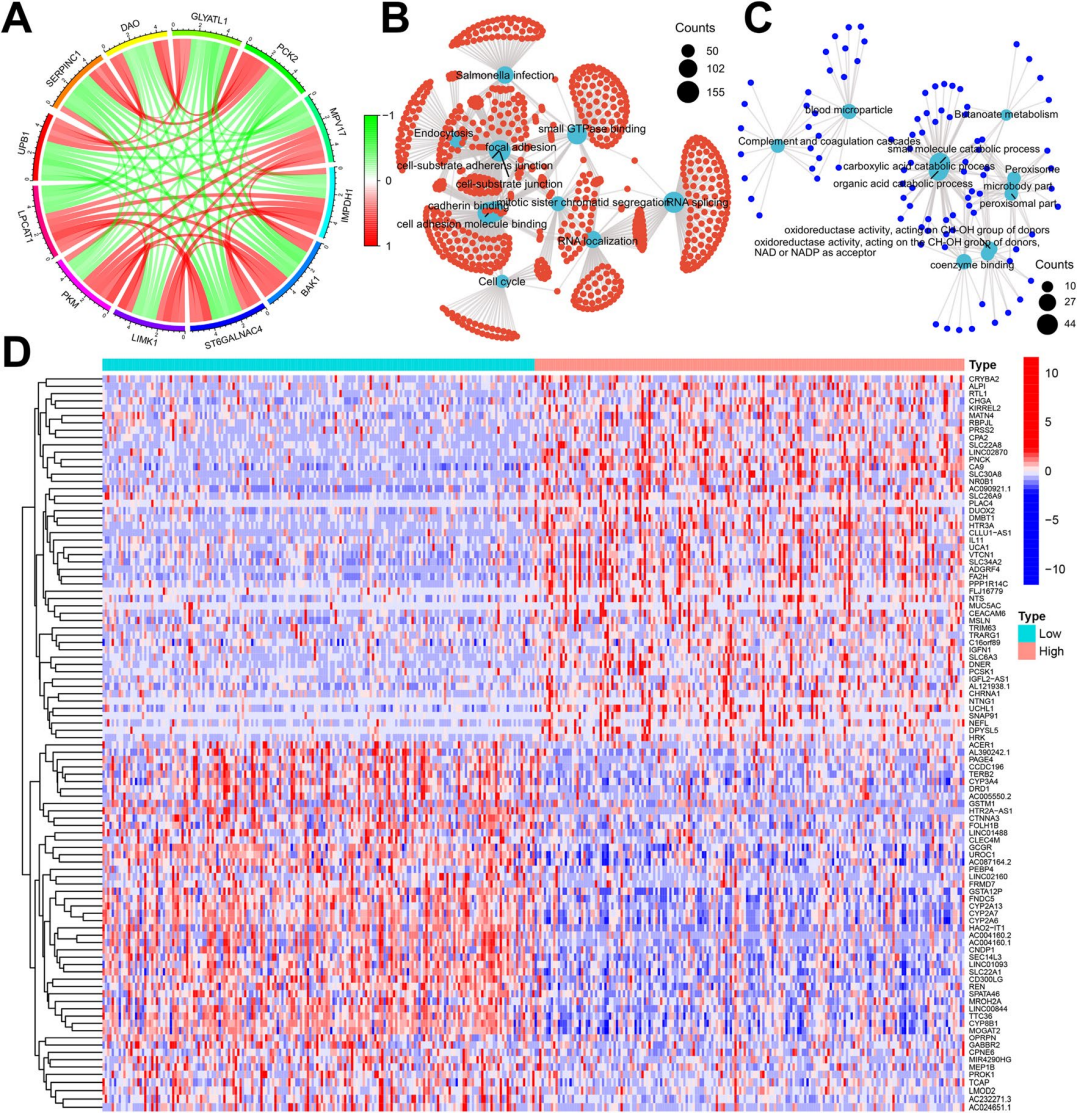
Analysis of the signaling pathway of LPCAT1 and its related genes. **A** Circle plot of top 5 genes positively (red) and negatively (green) correlated with LPCAT1 expression. GO and KEGG analysis of genes positively (**B**) or negatively (**C**) correlated with LPCAT1 expression in HCC. **D** Heatmap of the top 50 differentially expressed genes in high- or low-LPCAT1 groups

To further explore the biological significance of LPCAT1 in HCC, we performed a genome-wide gene profiling analysis of LPCAT1-related genes. Our results showed that there were 8702 genes associated with LPCAT1 expression (*p* < 0.01) in TCGA, and these significantly associated genes were classified as 7169 positively and 1533 negatively associated genes. Subsequently, we generated a circle plot of co-expression, as shown in Fig. 4A, the top 5 genes negatively correlated with LPCAT1 expression were UPB1, SERPINC1, DAO, GLYATL1 and PCK2, while the top 5 genes positively correlated with LPCAT1 expression were PKM, LIMK1, ST6GALNAC4, BAK1 and IMPDH1. To clarify which genes are differentially expressed between high- and low-LPCAT1 groups, we performed analysis and generated a heatmap of differential genes. As shown in Fig. 4D, the results present a heatmap of the top 50 differentially expressed genes that were most significantly up- and down-regulated in these two groups. GO and KEGG network analysis revealed that LPCAT1 positively associated molecules were mostly enriched in functions related to cell adhesion, cadherin binding, and cell cycle (Fig. 4B), while LPCAT1 negatively associated molecules were mostly enriched in functions related to metabolic process (Fig. 4C). These findings suggest that LPCAT1 may mediate cell–cell adhesion by altering the plasma membrane, thereby participating in multiple cancer-related signaling pathways, and ultimately promoting the development of HCC.

### Correlation between LPCAT1 and TME score in HCC

**Fig. 5:**
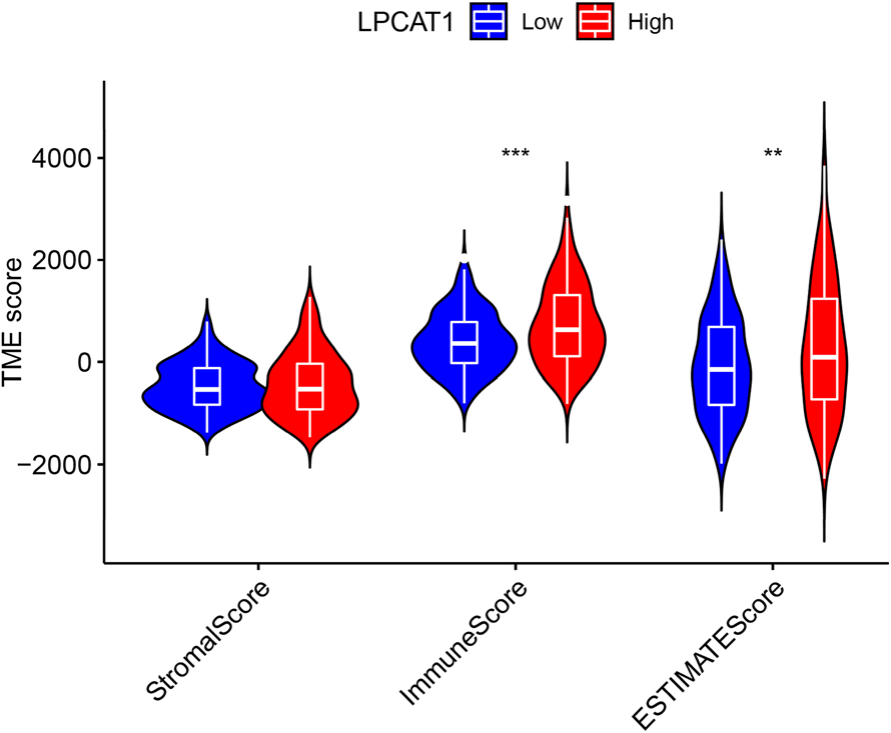
Correlation between LPCAT1 expression and TME score in HCC. *p < 0.05; **p < 0.01; ***p < 0.001

TME cells constitute a vital element of tumor tissue. Increasing evidence has elucidated their clinicopathological significance in predicting outcomes and therapeutic efficacy. We, therefore, analyzed the association of LPCAT1 expression with TME score in HCC. As shown in Fig. 5, the results showed that the TME score of high-LPCAT1 group was significantly higher than that of low-LPCAT1 group in HCC. It was further known that this difference was mainly due to differences in immune scores, not stromal scores.

### Correlation of LPCAT1 expression with immune cell infiltration in HCC

**Fig. 6:**
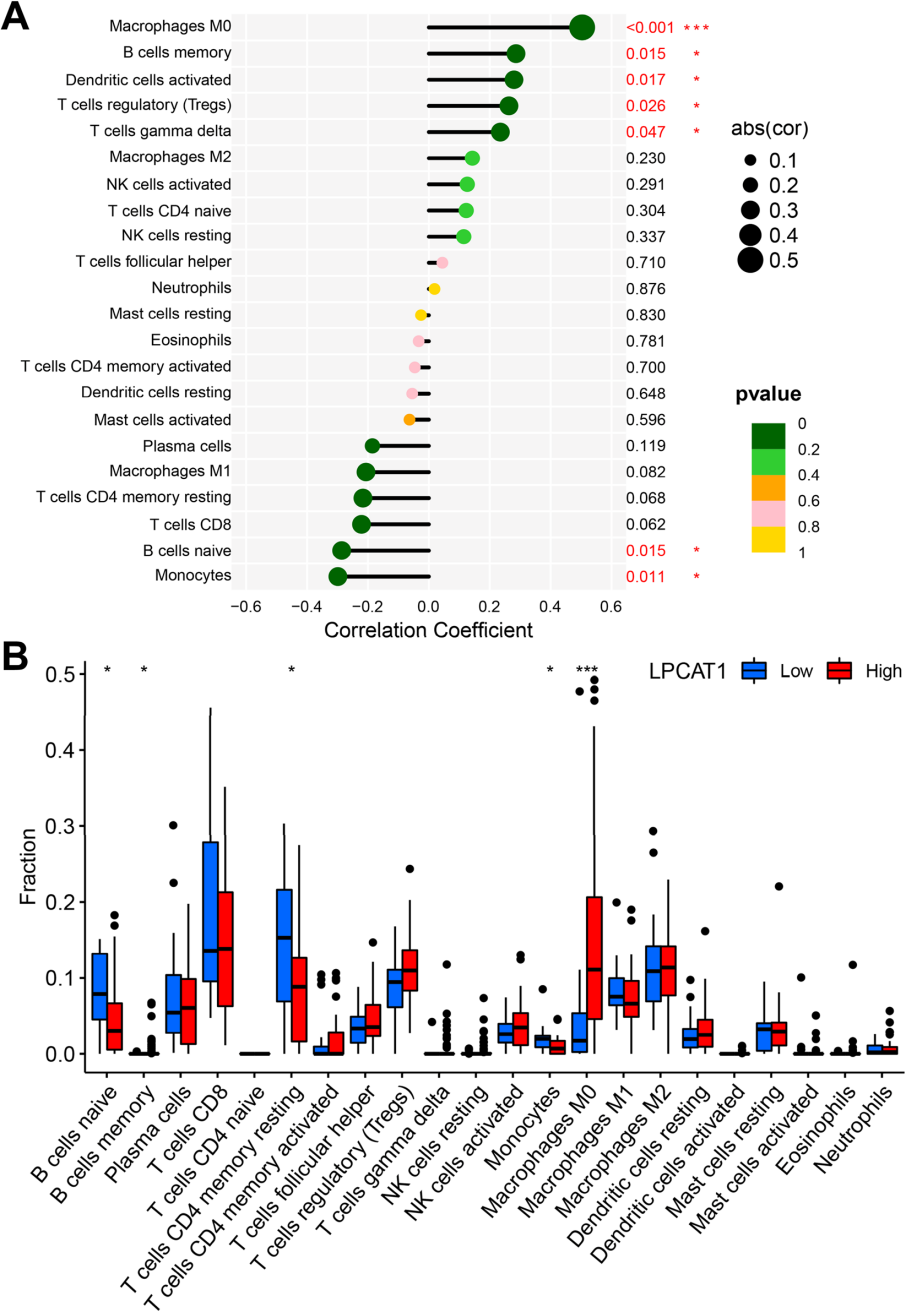
Correlation between LPCAT1 expression and immune cell infiltration in HCC. **A** Correlation of LPCAT1 expression level with various immune cell infiltration level in HCC. **B** Differences in the infiltration of various immune cells within high- and low-LPCAT1 groups. *p < 0.05; **p < 0.01; ***p < 0.001

LPCAT1 is thought to play an important role in the immune system, so we explored the correlation between LPCAT1 expression and immune cell infiltration. As shown in Fig. 6A, we found that the immune infiltrating cells positively correlated with LPCAT1 expression were Macrophages M0 (*p* < 0.001), B cells memory (*p* = 0.015), Dendritic cells activated (*p* = 0.017), T cells regulatory (*p* = 0.026) and T cells gamma delta (*p* = 0.047), while the immune infiltrating cells negatively correlated with LPCAT1 expression were B cells naive (*p* = 0.015) and Monocytes (*p* = 0.011) in HCC. Next, we analyzed the differences in the infiltration of various immune cells within high- and low-LPCAT1 groups. As shown in Fig. 6B, the results showed that the infiltration levels of B cell naive and T cells CD4 memory resting in high-LPCAT1 group were significantly lower than those in low-LPCAT1 group. Differently, the infiltration level of Macrophages M0 in high-LPCAT1 group was significantly higher than that in low-LPCAT1 group. Our results demonstrated that LPCAT1, together with immune cells, are involved in the progression of HCC.

### Correlation between LPCAT1 and immune checkpoint gene expression in HCC

**Fig. 7:**
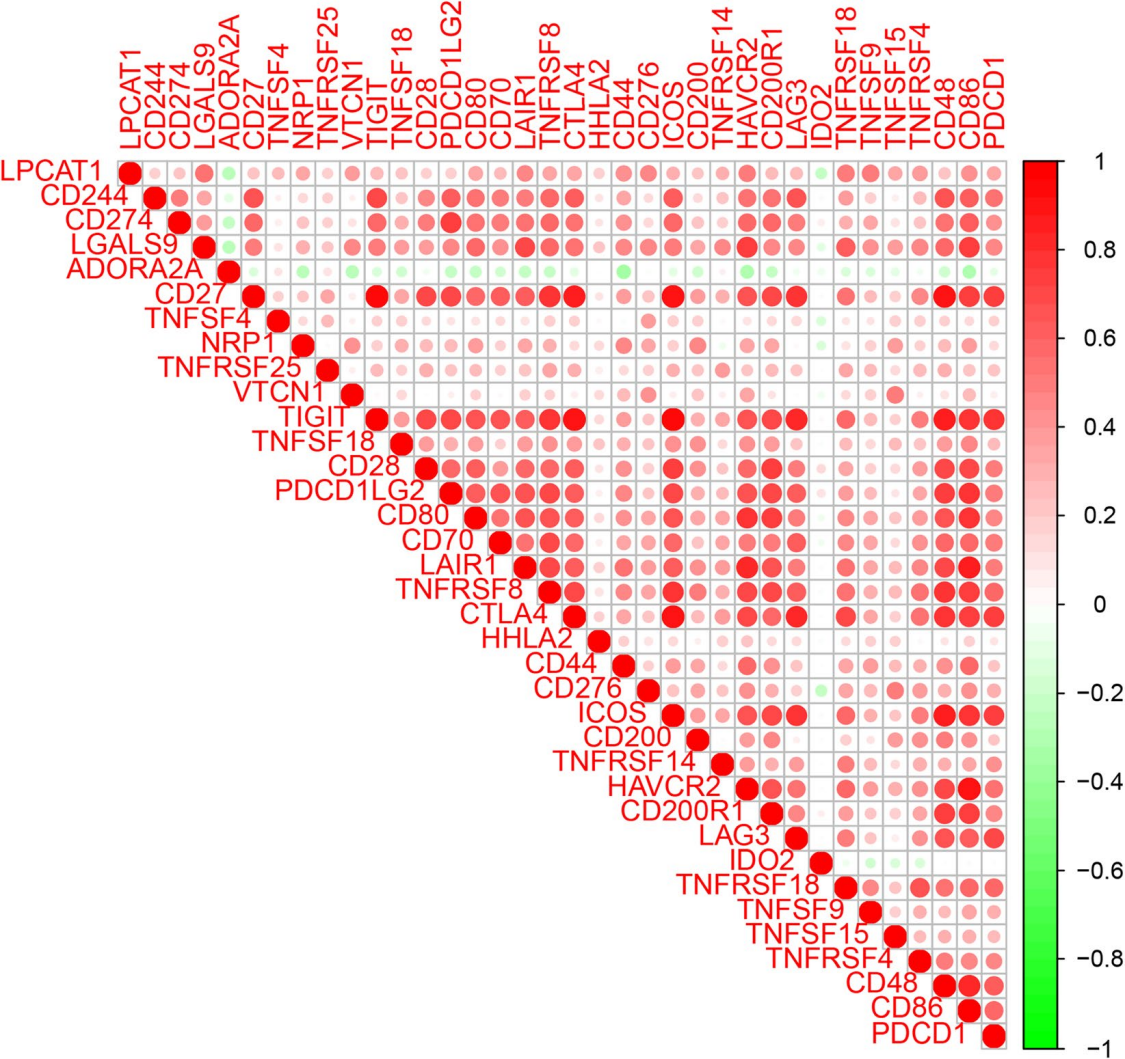
Correlation between LPCAT1 expression and immune checkpoint gene expression in HCC

It is well-known that immunotherapy plays an important role in HCC, and the expression of immune checkpoint genes can not only predict prognosis, but also predict the response to immunotherapy. We, therefore, analyzed the correlation between LPCAT1 expression and immune checkpoint gene expression. As shown in Fig. 7, LPCAT1 was positively correlated with almost all immune checkpoint genes, including LGALS9, LAIR1, HAVCR2, TNFRSF18, TNFRSF4, TNFSF9, CD86, CD276, CD44, VTCN1, CD80, TNFRSF8, CTALA4, NRP1, ICOS, PDCD1, and TNFRSF14, while LPCAT1 was negatively correlated with ADORA2A and IDO2. We found a positive correlation between LPCAT1 and most immune checkpoint gene expression, suggesting that LPCAT1 can be used as one of the biomarkers for predicting immunotherapy response.

### Correlation between LPCAT1 expression and drug sensitivity in HCC

**Fig. 8:**
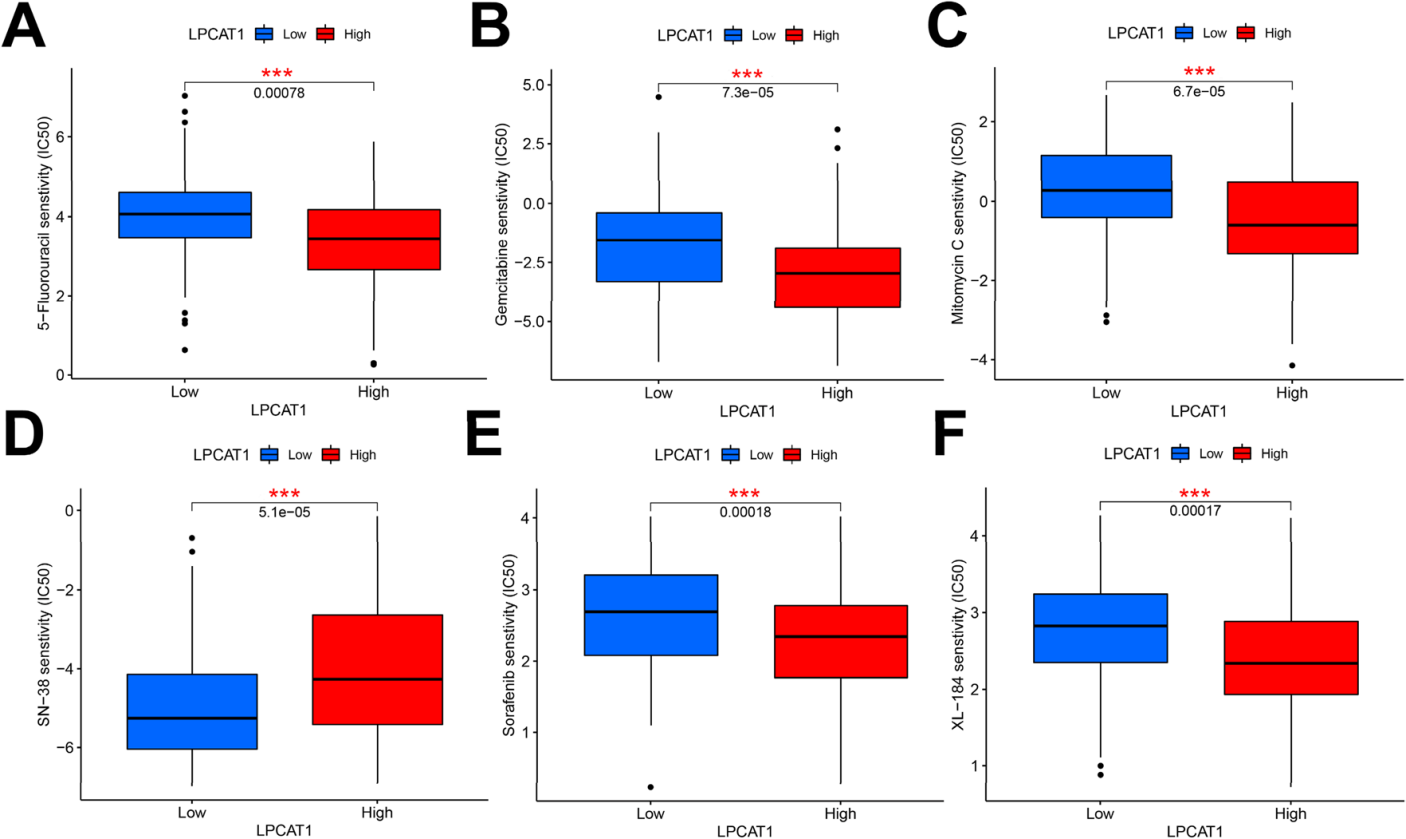
Correlation between LPCAT1 expression and drug sensitivity in HCC, including 5-Fluorouracil (**A**), Gemcitabine (**B**), Mitomycin C (**C**), Irinotecan (**D**), Sorafenib (**E**), and Cabozantinib (**F**). *p < 0.05; **p < 0.01; ***p < 0.001

To explore the relationship between LPCAT1 and drug sensitivity, we performed a drug sensitivity analysis within high- and low-LPCAT1 groups. As shown in Fig. 8, we screened out the sensitivity of 6 drugs related to HCC treatment in total. Among them, the IC50 of 5-Fluorouracil, Gemcitabine, Mitomycin C, Sorafenib and Cabozantinib (XL-184) in patients with high-LPCAT1 expression was lower than that in patients with low-LPCAT1 expression, indicating a better efficacy in high-LPCAT1 expression group. and the IC50 of Irinotecan (SN-38) in patients with low-LPCAT1 expression was lower than that in patients with high-LPCAT1 expression, indicating a better efficacy in low-LPCAT1 expression group. This suggests that LPCAT1 may be involved in the resistance to these drugs in HCC.

## Discussion

Metabolic pathways, including lipid metabolism, are frequently reprogrammed within cancer cells to adapt to environmental challenges and promote their expansion and metastasis [3]. As an important enzyme in phosphatidylcholine metabolism, LPCAT1 can affect the energy metabolism of tumors by modulating the level of saturated phosphatidylcholine and changing the phospholipid composition of the plasma membrane [11]. LPCAT1 has been shown to be associated with various cancers and play an important role in the occurrence and development of various tumors [17–21]. Our results indicate that LPCAT1 is overexpressed in most human tumors, suggesting that LPCAT1 may play a key promoting role in the development of these cancers including HCC. Further prognostic analysis demonstrated high-LPCAT1 expression was associated with poor prognosis in HCC. Further univariate/multivariate analysis confirmed the prognostic value of LPCAT1 expression in HCC and as an independent prognostic biomarker for patients with HCC.

LPCAT1 expression correlates with clinicopathological features in HCC. Zhang found that LPCAT1 expression was related to tumor grade, ECOG score, AFP and TNM stage [19, 22]. We analyzed and found more relationships between LPCAT1 and clinicopathological features. Our study not only found and confirmed the correlation between LPCAT1 expression and more clinicopathological features in HCC, and further found that LPCAT1 expression was positively correlated with the risk of hepatocellular carcinoma, which could well reflect the clinical staging and grading of HCC patients.

**Fig. 9:**
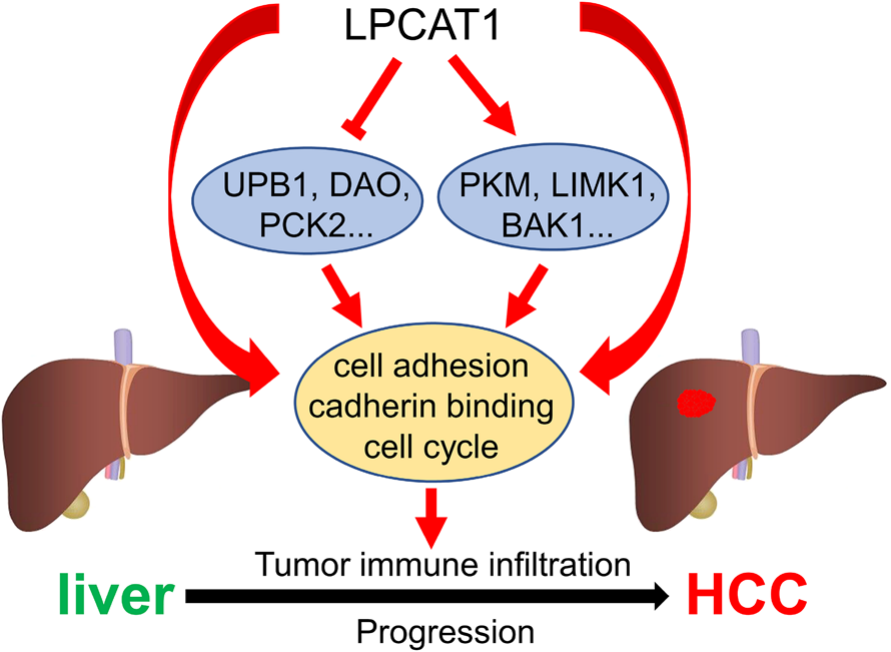
Schematic representation of the putative role of LPCAT1 in hepatocellular carcinoma

There have been several studies on the mechanism of LPCAT1 in tumor cells. Wei demonstrated that LPCAT1 was up-regulated in NSCLC cells and tissues, and promoted NSCLC progression via PI3K/AKT/MYC pathway, can be used as a target for the NSCLC treatment [23]. LPCAT1 was found to contribute to cutaneous squamous cell carcinoma progression through EGFR-mediated protein kinase B and p38MAPK signaling pathways [24]. High-LPCAT1 expression could inhibit STAT1 expression, up-regulate CyclinD1, CyclinE, CDK4 and MMP-9, and decrease p27kip1 to promote cancer progression in HCC [25]. More knowledge is needed to understand how LPCAT1 promotes tumorigenesis in HCC. Our results revealed genes positively associated with LPCAT1 are closely associated with numerous biological processes, including regulation of cell cycle, actin filament organization, Ras protein signal transduction, autophagy and regulation of apoptotic signaling pathway. We further found that LPCAT1 positively related genes are associated with many cancer-related signaling pathways, including VEGF, Rap1, Notch, Hippo, Apoptosis, p53, TNF and HIF-1. GO and KEGG network analysis revealed that LPCAT1 positively associated molecules were mostly enriched in functions related to cell adhesion. These findings suggested that LPCAT1 may be involved in multiple cancer-related signaling pathways by mediating intercellular adhesion and ultimately contributing to the development of HCC (Fig. 9).

Immunotherapy has increasingly become an important part of the treatment of tumor therapy [26, 27]. The sensitivity of immunotherapy for HCC directly determines the prognosis, which is very critical. We further explored the relationship between LPCAT1 expression and TME score, immune-infiltrating cells, immune checkpoint gene expression and drug sensitivity. Tumor-infiltrating immune cells are thought to be involved in tumor proliferation and metastasis [28–32]. We studied the correlation of LPCAT1 expression with the infiltration status of immune cells in HCC and found LPCAT1 expression was closely correlated with immune cell infiltration in HCC. Immune infiltrating cells positively correlated with LPCAT1 expression were Macrophages M0, B cells memory, Dendritic cells activated, T cells regulatory and T cells gamma delta, while the immune infiltrating cells negatively correlated with LPCAT1 expression were B cells naive and Monocytes, suggesting that LPCAT1 plays a role in regulating tumor immunity in hepatocarcinogenesis. The infiltration levels of B cell naive and T cells CD4 memory resting in high-LPCAT1 group were significantly lower than those in low-LPCAT1 group. Differently, the infiltration level of Macrophages M0 in high-LPCAT1 group was significantly higher than that in low-LPCAT1 group. Furthermore, LPCAT1 expression was found to be positively correlated with the expression of exhausted T cells markers (CTL-4 and PD-1), which are key immune checkpoints used by cancer cells to help evade immune surveillance. The induction of immune cells into the tumor by immune checkpoint blockade is the key to tumor immunotherapy [33]. Thus, the effectiveness of immunotherapy depends primarily on sufficient immune cell infiltration and adequate expression of immune checkpoints in the tumor microenvironment [34]. Finally, we explored the relationship between LPCAT1 expression and drug sensitivity, and found that LPCAT1 can be used as a biomarker to predict the efficacy of some drugs, including 5-Fluorouracil, Gemcitabine, Mitomycin C, Sorafenib, Cabozantinib and Irinotecan in HCC. This helps clinicians make more scientific decisions in HCC treatment. And also, it is suggested that LPCAT1 may be involved in the resistance of these drugs and is associated with treatment resistance [35]. Han found LPCAT1 overexpression led to castration resistant prostate cancer cell resistance to treatment with paclitaxel. All our findings together illustrated that LPCAT1 functions as a regulator in tumor-infiltration of immune cells in HCC, and targeting LPCAT1 may improve the efficacy of immunotherapy in HCC.

## Conclusions

In conclusion, we clarified that LPCAT1 is an independent prognostic biomarker associated with TME score, immune cell infiltration, immune checkpoint gene expression, which can well reflect the clinical stage and grade of HCC patients. LPCAT1 is associated with drug sensitivity, which provides support for oncologists to formulate anti-HCC treatment strategies. At last, our findings provide a wealth of information for further understanding of the biological functions and signaling pathways of LPCAT1 in HCC.

## Data Availability

The data sets generated and/or analyzed during the current study are available from the corresponding author on reasonable request.

## Abbreviations

LPCAT1: Lysophosphatidylcholine acyltransferase 1
HCC: Hepatocellular carcinoma
TCGA: The cancer genome atlas
THPA: The human protein atlas
GO: Gene ontology
KEGG: Kyoto encyclopedia of genes and genomes
OS: Overall survival
PFS: Progression-free survival
NSCLC: Non-small cell lung cancer
BLCA: Bladder urothelial carcinoma
BRCA: Breast invasive carcinoma
CESC: Cervical squamous cell carcinoma and endocervical adenocarcinoma
CHOL: Cholangiocarcinoma
COAD: Colon adenocarcinoma
ESCA: Esophageal carcinoma
GBM: Glioblastoma multiforme
HNSC: Head and neck squamous cell carcinoma
KICH: Kidney chromophobe
KIRC: Kidney renal clear cell carcinoma
KIRP: Kidney renal papillary cell carcinoma
LIHC: Liver hepatocellular carcinoma
LUAD: Lung adenocarcinoma
LUSC: Lung squamous cell carcinoma
PAAD: Pancreatic adenocarcinoma
PCPG: Pheochromocytoma and paraganglioma
PRAD: Prostate adenocarcinoma
READ: Rectum adenocarcinoma
STAD: Stomach adenocarcinoma
THCA: Thyroid carcinoma
UCEC: Uterine corpus endometrial carcinoma
